# Computer Controlled Automated Assay for Comprehensive Studies of Enzyme Kinetic Parameters

**DOI:** 10.1371/journal.pone.0010727

**Published:** 2010-05-19

**Authors:** Felix Bonowski, Ana Kitanovic, Peter Ruoff, Jinda Holzwarth, Igor Kitanovic, Van Ngoc Bui, Elke Lederer, Stefan Wölfl

**Affiliations:** 1 Department of Biology, Institute for Pharmacy and Molecular Biotechnology, Ruperto-Carola University of Heidelberg, Heidelberg, Germany; 2 Centre for Organelle Research, Faculty of Science and Technology, University of Stavanger, Stavanger, Norway; Dana-Farber Cancer Institute, United States of America

## Abstract

Stability and biological activity of proteins is highly dependent on their physicochemical environment. The development of realistic models of biological systems necessitates quantitative information on the response to changes of external conditions like pH, salinity and concentrations of substrates and allosteric modulators. Changes in just a few variable parameters rapidly lead to large numbers of experimental conditions, which go beyond the experimental capacity of most research groups. We implemented a computer-aided experimenting framework (“robot lab assistant”) that allows us to parameterize abstract, human-readable descriptions of micro-plate based experiments with variable parameters and execute them on a conventional 8 channel liquid handling robot fitted with a sensitive plate reader. A set of newly developed R-packages translates the instructions into machine commands, executes them, collects the data and processes it without user-interaction. By combining script-driven experimental planning, execution and data-analysis, our system can react to experimental outcomes autonomously, allowing outcome-based iterative experimental strategies. The framework was applied in a response-surface model based iterative optimization of buffer conditions and investigation of substrate, allosteric effector, pH and salt dependent activity profiles of pyruvate kinase (PYK). A diprotic model of enzyme kinetics was used to model the combined effects of changing pH and substrate concentrations. The 8 parameters of the model could be estimated from a single two-hour experiment using nonlinear least-squares regression. The model with the estimated parameters successfully predicted pH and PEP dependence of initial reaction rates, while the PEP concentration dependent shift of optimal pH could only be reproduced with a set of manually tweaked parameters. Differences between model-predictions and experimental observations at low pH suggest additional protonation-sites at the enzyme or substrates critical for enzymatic activity. The developed framework is a powerful tool to investigate enzyme reaction specifics and explore biological system behaviour in a wide range of experimental conditions.

## Introduction

Lab automation systems generate large amounts of experimental data in a short amount of time and substantially reduce costs per data-point. The elimination of manual bench-work does not only minimize time and expenses, it also provides very homogenous datasets that facilitate comparison and the application of statistical methods.

In the recent years, robotic systems have been successfully used to automate a number of important experiments in biopharmaceutical research such as screening and synthesis of drug candidates [1], genome wide RNAi screens [2], DNA sequencing [3] and elucidation of protein-protein interactions [4]. While high-throughput screens are almost exclusively carried out using some kind of automated laboratory equipment, the development of new methods and the in depth investigation of selected biological systems has not profited from the benefits of automation to the same extent yet. Implementing a protocol on an automated system can be a tedious and time consuming task, and in a setting with constantly changing experimental procedures, the time needed for adapting programs often renders lab automation systems unattractive. In order to be usable for changing experimental setups in a science lab, they have to be flexible and must provide more benefits than just a simple reduction of repetitive pipetting work. One such benefit can lie in the ability of a computer-controlled lab-automation-system to keep track of sample identities and reagent volumes. This allows the use of complicated, computer generated, experimental designs described by hundreds of decimal numbers that would be difficult or even impossible to implement manually. If data acquisition and analysis is integrated with experimental design and execution, the lab-automation-system can react to experimental outcomes immediately after the measurement by modifying experimental parameters and carrying out the additional experiments autonomously. As the connection between sample identities, experimental parameters and measurement results is retained during the whole process, data-analysis and interpretation is greatly facilitated.

The fully automated “Robot Scientist” demonstrated in [5] is a particularly advanced example of a system for quantitative phenotypic analysis that integrates all steps of the scientific process, from hypothesis creation, through testing the hypothesis and results interpretation to the planning of further experimental steps based on the obtained results. While being an impressive tool for next generation high-throughput experiments, such a system is still expensive and not suitable for the relatively small batch sizes and constantly changing protocols encountered in the daily work of characterizing biological entities and developing new methods.

In this work, we describe a framework that was designed to bride the gap between advanced high-throughput systems and manual bench work. It integrates automated protocol execution with computer-based data interpretation to allow reactions to measurement outcomes in autonomous experiments, but at the same time it has been designed to make it as easy as possible to use it for different types of experiments without extensive reprogramming.

Our system is unique by the fact that it is based on abstract, object oriented descriptions of experiments written in the R [6] programming language that provides a wide range of statistical tools for experimental planning and data-analysis. It allows to carry-out experiments by specifying only abstract protocol parameters such as component names, concentrations and incubation times. The necessary calculations for translating this information into volumes, pipetting patterns and commands that can be executed by a robot are done automatically, greatly reducing the effort for setting up new experiments and providing a very convenient interface to computer-based experiment planning.

We implemented a variety of tools for generating experimental designs, optimizing experimental procedures and for analyzing, visualizing and interpreting experimental data. All of them interact seamlessly with the automation framework, avoiding unnecessary data-conversions and allowing their use in autonomous experiments.

We applied the newly developed methods mentioned above on a variety of biochemical systems and present a few examples of how it can be used to collect the data for solving diverse scientific problems. We used the system for optimizing buffer mixtures of assays for determining the activity of several glycolytic enzymes. By automatically testing systematic variations of the known standard-protocols, we were able to determine buffer mixtures that substantially increase the activity of the enzymes of interest when compared to the protocols commonly found in literature (data not shown).

One of the glycolytic enzymes, pyruvate kinase (PYK), was investigated in more detail. After an initial experiment in which we investigated the interplay of multiple different modulators of enzyme activity, we became interested in the pH dependence of enzymatic activity. We acquired data for a model of pH dependence of enzyme activity, determined its parameters by nonlinear regression and compared its predictions with our measurement results.

## Results

### 1. Optimization of buffer-mixtures for enzymatic activity assays

In a setting where quantitative experimental parameters have to be varied in order to achieve an optimization goal that can be expressed in a single number, numerical methods allow to get closer to an optimal set of parameters in a systematic manner. Numerical optimization methods typically treat the relation between input parameters and experimental outcome as a black box function that satisfies a few general criteria like smoothness [7], [8]. Therefore, they can be applied even when no valid mathematical model of the process under investigation is known. In order to demonstrate how our framework can be used to find optimal reaction conditions for enzymatic assays in an automated iterative procedure, we performed an optimization of pH, KCl and Fructose-1,6-bisphosphate concentrations for maximum PYK activity in five rounds of experiments. In the first round, we measured the activity of the enzyme in 20 different mixtures that were chosen according to a space filling experimental design that covered the complete allowed concentration/pH range of all three variable components. The data from this experiment was used to build an initial Kriging model of the concentration and pH dependence of PYK activity.

In each of the remaining four rounds, the response-surface model was refined with 19 additional measurements. The parameters of these measurements were selected by our algorithm to lie in those regions where the lower bound of the model estimate is lowest, while keeping a user-specified minimum distance to other measurements (see methods section).

The minimum distance between points in the first refinement round was set to 0.25 with all parameters normalized to a [0,1] interval. After each round, the minimum distance was multiplied with 2/3 in order to allow a finer sampling of the area around the estimated optimum. After each round of measurements, the response-surface was updated with the new results and its optimum was determined using R's built in optimization method. The complete experiment was carried out autonomously without user interaction and ran for about two hours.

**Figure 1:**
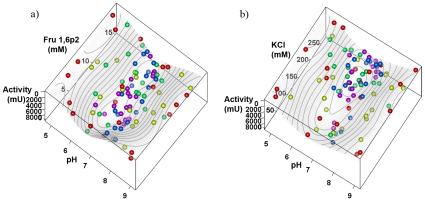
The final response-surface model and the measurements of the five optimization rounds (red, yellow, green, blue and purple). Data are presented as enzyme activity dependence on (a) Fru1,6P2 concentration and pH or (b) KCl concentration and pH. The interpolated response surface correspond to a slice of third variable (KCl in (a); Fru1,6P2 in (b)) at optimal condition. Note how the measurements of the later iterations cluster around the optimum, providing additional information about this region.

Fig. 1 shows the measurements of the five iterations and the final response-surface model plotted together. The measurements of the later iterations are clustered around the optimum of the final response-surface model, providing much more information about this region than 96 evenly distributed measurements could. The optimal parameters estimated after the first iteration already lay close to the centre of this region, showing that the algorithm is capable of finding useful reaction parameters even from minimal number of experimental conditions tested if the range of conditions is selected correctly.

The quality of the optimum estimate is somewhat hard to assess objectively because the true location of the optimum is unknown and the measurements are subject to a substantial amount of noise. To get some benchmark of the quality of the optimum in the response-surfaces for the individual iterations, we selected the five measurements closest to the estimated optimum and averaged their measured activities. The results in Table 1 show the activity of measurements close to the optimum increases from iteration to iteration, reflecting an improvement in the quality of the estimate.

**Table 1:** Optimal condition estimates and average activity of the five measurements whose parameters are most similar to them over the five iterations.

Iteration		1	2	3	4	5
Estimated Optimum	Fru-1,6p2 (mM)	8.70	9.24	10.88	10.90	12.28
	KCl (mM)	253.96	188.43	229.19	204.61	200.41
	pH	7.00	6.92	6.76	6.86	6.81
Mean activity of 5 measurements closest to optimum estimate (mU)		6438	6660	7527	7648	7680

Notice how the activity of measurements close to the optimum increases from iteration to iteration, reflecting an improvement in the quality of the estimate.

### 2. An investigation of the influence of pH, Fructose 1,6-bisphosphate (fru 1,6-p_2_) and pho(enol)pyruvate (PEP) on the enzymatic activity of PYK

**Figure 2:**
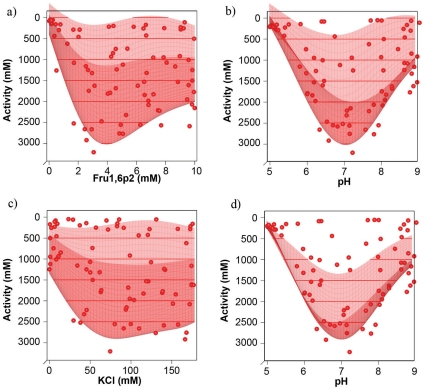
KCl, Fructose 1,6-bisphosphate and pH dependence of PYK activity. Each dot corresponds to a measurement conducted with one of 80 conditions from the space filling design of the first round of the experiment described in 3.2. The four plots show two slices along different axes of the parameter space from two different perspectives. In plot a) and b) the KCl concentration is held fixed at its optimum of 90.6 mM while Fructose 1,6-bisphosphate concentration and pH are variable, in plot c) and d), the Fructose 1,6-bisphosphate concentration is held fixed at its optimum of 4.1 mM with variable KCl concentration and pH. In all plots, the corresponding slice through the Kriging model fitted to the data is shown as a surface.

The stability of biomolecules and the ability of proteins to perform their specific tasks is highly dependent on their physicochemical environment. In order to survive, living organisms maintain internal conditions that stabilize their underlying structures and allow the right biochemical reactions to take place. The ability of an enzyme to perform its task is influenced by the composition of its environment, like availability of certain ions and metals or presence of allosteric inhibitors or activators. To illustrate how our automated computer controlled framework can be used to investigate the influence of the physicochemical environment on enzyme activity, we investigated the effects of KCl, pH and Fructose 1,6-bisphosphate on PYK activity while keeping substrate (PEP) concentration constant at 1mM. We first measured the activity of PYK in 80 different mixtures that were chosen according to a space filling experimental design that covered the complete allowed concentration/pH range of all three variable components. Fig. 2 shows the measured data-points and two different slices of a Gaussian Random Process Regression model fitted to the data.

It can be seen that all three variables strongly influence enzymatic activity. The optimum conditions determined from this model are KCl 90.6 mM, Fructose 1,6-bisphosphate 4.1 mM and pH 7.06.

Enzymatic activity decreases quickly when deviating more than half a pH unit from the optimum pH either to the acidic or basic side. Sufficiently high salt concentration is needed for the enzyme activity – the activity drops quickly at KCl concentrations below 50 mM. High salt concentrations also inhibit the enzyme, but to a lesser extent. Fructose 1,6-bisphosphate is known as an allosteric activator that influences the K_M_ of pyruvate kinase by changing its conformation when binding to it [9], [10]. Enzymatic activity at the substrate concentration used in this experiment decreases strongly if the Fructose 1,6-bisphosphate concentration is below 3 mM. Interestingly, high concentrations of Fructose 1,6-bisphosphate inhibit the enzyme as well. This behaviour has been reproduced in multiple similar experiments and, to our knowledge, has not been described in literature until now.

**Figure 3:**
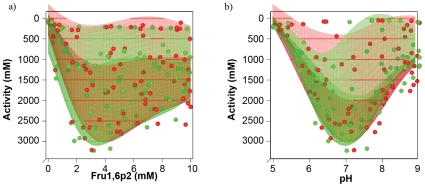
Comparison of PYK activities determined with fixed and variable KCl concentrations. a) and b) show two different perspectives of the same plot. The data shown in red is from the first round of the experiment described in 3.2 and identical to that shown in Fig. 9. The data in green was measured directly at the KCl concentration of the slice of the Kriging model from round one. The fit-surfaces of two datasets are very similar, indicating that the interpolation from the variable KCl model gives a good prediction of the activities at the optimal KCl concentration.

We validated the quality of our response-surface models in a second experiment in which we kept KCl concentration fixed at 90.6 mM while varying pH values and Fructose 1,6-bisphosphate concentrations in eighty mixtures according to a space-filling design. The data obtained from this experiment was compared to the activities predicted for this KCl concentration by the first model as an indicator of its predictive strength. Both datasets and the models based on them are shown together in Fig. 3. Although not completely identical, the models constructed form the two different data-sets are very similar, indicating that the interpolation from data points with different KCl concentrations provided a reasonable estimate of the activity at 90.6 mM KCl.

In order to analyze the influence of substrate concentration on the pH dependence and allosteric modulation of PYK, we performed an experiment in which we tested five different PEP concentrations (0.25, 0.5, 1, 2 and 4 mM) for each of the 17 points of a space filling design with variable pH (4.75–9.2) and Fructose 1,6-bisphosphate (0–10 mM).

**Figure 4:**
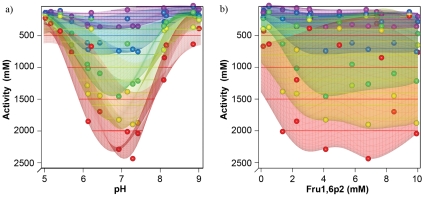
Influence of pH and Fructose 1,6-bisphosphate on PYK activity at different substrate (PEP) concentrations. a) and b) show two different perspectives of the same plot. Each colour represents one PEP concentration. 0.25 mM is shown in red, 0.5 mM in yellow, 1 mM in green, 2 mM in blue and 4 mM in purple. Note the complex pattern of interactions and how the pH optimum shifts toward a more acidic pH at low substrate concentrations. Due to the relatively low number of measurements, it is not completely possible to exclude fitting artefacts as a source of variability of the fitted surfaces.

The 95 different activities determined in the experiment were used to construct a Gaussian Random Process Regression surface with logarithmic scaling of the PEP and Fructose 1,6-bisphosphate axes for visualization. Fig. 4 shows the complex interaction of the variable factors. The data indicates that the pH optimum of pyruvate kinase depends on the PEP concentration with a shift toward acidic pH at low substrate concentrations. The effect was reproduced in multiple experiments, but the number of data-points spread out over the three-dimensional parameter space was not high enough to quantify the exact changes of the optimal pH-value.

### 3. Modelling of PYK activity dependence on pH

Systematic measurements of enzymatic activity at different pH-values and substrate concentrations can help to reveal mechanistic details of substrate binding and the enzymatic reaction-steps involved. In such a context, our framework is ideally suited to provide large homogenous datasets for parameterizing and validating quantitative models.

We investigated the pH dependence of PYK behaviour in a dedicated experiment. The set of measurements consisted of combinations of pH values (4.75, 5.39, 6.02, 6.66, 7.29, 7.93, 8.56 and 9.2) with PEP concentrations of (0.25, 0.5, 1, 2, 4 and 8 mM) with two replicates per combination, resulting in a total of 96 measurements. The Fructose 1,6-bisphosphate concentration was held fixed at 3.5mM, a value found to be sufficient for PYK activation in the previous series of experiments.

**Figure 5:**
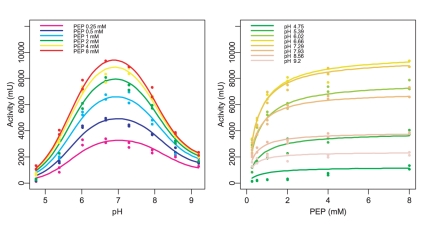
Dependence of PYK activity on pH and PEP concentration. In graph a), each colour corresponds to a slice through the parameter space with a fixed PEP concentration, in graph b), each colour corresponds to a slice with a fixed pH value. The dots represent measured data, the straight lines show the predictions of the diprotic model fitted to the data. The measured data indicates a shift of the pH optimum towards more acidic pH values at low substrate concentrations, but the fitted model does not show this shift.

The parameters for a diprotic model of pH-dependence of PYK activity were determined from the measured data in a multistart least squares procedure as described in methods. A comparison of the measured data and the predictions given by the diprotic model is shown in Fig. 5. The diprotic model with the set of parameters we determined successfully predicts the influence of pH and initial PEP concentration on the initial reaction rates at intermediate pH values, but it does not show the PEP concentration dependent shift in optimum pH that is visible in the measurements. In addition, it fails to describe the substrate-concentration dependence at the lowest pH value in the experiment, pH 4.75. Despite extensive efforts, a single set of parameters that predicts all features visible in the measured data at the same time could not be found.

**Figure 6:**
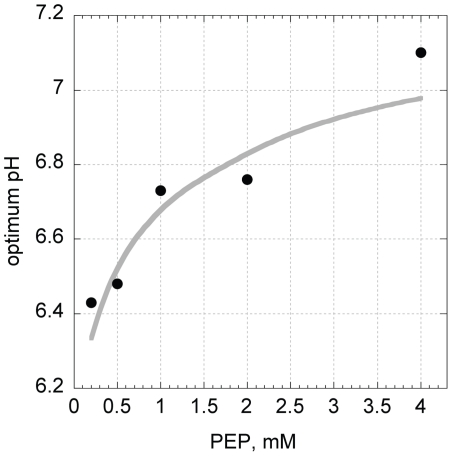
Optimum pH as a function of initial substrate concentration. Solid grey line shows the calculated optimum pH (Eq. 5) using a set of manually determined parameters. Solid dots show experimental results.

The substrate-concentration dependent shift of the optimal pH can be described by a diprotic model with a set of hand-fitted parameters that satisfies K1E*K2E≫K1ES*K2ES, but such a solution does not minimize the sum of squared residuals. Fig. 6 shows a comparison of the observed pH-shifts and the predictions of the hand-fitted model.

**Figure 7:**
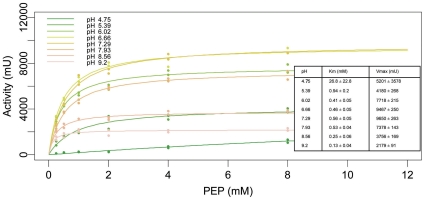
Substrate dependence of PYK activity at different pH-values and fitted Michaelis Menten curves. The small table in the figure shows the Michaelis Menten constants and their estimated uncertainties as determined by the fitting procedure. Note the pH dependent trend in Km and the drastically lower substrate-affinity at pH = 4.75.

**Figure 8:**
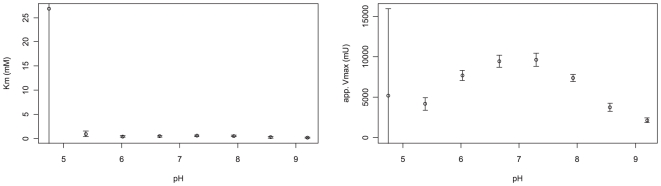
A graphical representation of the apparent Km and Vmax-values determined at different pH. Note the shape of the pH-dependence of Vmax and the high reported uncertainties at pH 4.75.

For a more detailed analysis, we determined the coefficients of a Michaelis-Menten-Kinetic [11] for each pH value in the experiment (see Fig. 7, 8). In the range from pH 5 to pH 9.2, the apparent Km and Vmax values vary relatively smoothly, with a clear Vmax optimum in the neutral pH range and a steady decrease of Km from Km = 0.94mM at pH 5.39 to Km = 0.12 at pH 9.2. At pH 4.75, the measured activities did not reach a substrate saturation plateau (see Fig. 7). As the substrate-concentration dependence of reaction speed in the measured range was effectively first-order, it was impossible to determine a meaningful set of Michaelis-Menten constants for pH 4.75. The uncertainty of Km at pH 4.75 reported by the fitting procedure was extremely high, but manual inspection of the residuals at different Km values (data not shown) suggested a Km well above 4mM, a drastic change from the Km = 0.94 mM at pH 5.39.

## Discussion

Combining the abstractive power and extensibility of established object oriented programming environments with existing lab-automation hardware opens up a wide range of new applications beyond conventional high-throughput screens. It greatly reduces the amount of programming necessary for performing new variants of similar experiments and thereby increases the usefulness of automated systems for small- to mid-scale experiments such as the development of new methods and the exploration biological system behaviour.

All these experiments took only between one and two hour to carry out after the source stocks had been prepared. The experiments on pH, KCl and Fructose 1,6-bisphosphate dependence of PYK activity show how automated experimenting can be used to gain quantitative understanding of the interplay for many modulators of enzymatic activity that are present in the intracellular environment.

In the context of systems biology, computer controlled experimenting as we implemented provides an extremely effective way of generating the large amounts of quantitative data needed for parameterizing and validating complicated models with numerous parameters.

A single two-hour experimental run was sufficient to generate sufficient data to determine the eight unknown parameters of the diprotic model we used to model the pH and substrate concentration dependence of PYK activity. The large number of data points helps to prevent overfitting and reveal insufficiencies in the model. The dataset we used for determining the model parameters shows a complex pattern of pH dependence of substrate affinity that cannot be fully explained by a simple diprotic model. The differences between model-predictions and observations in the low pH-range suggest that additional protonation-sites at the enzyme or protonation of the substrates ADP and PEP would have to be included into the model in order to make it more realistic. In a smaller or less homogenous dataset, these differences would have been much harder to notice.

Most experimental restrictions of our system are posed by the hardware we have to our disposal: Experimental procedures are limited to pipetting, plate transfers and photometric measurements and the dynamic range and number of components in the mixtures is limited by pipetting precision. If a large number of components are needed in a mixture, their individual volumes become so small that there is little room for variation without reducing them to values that are close to the absolute pipetting error. In the future, we plan to include an automatic handling of different stock concentrations to alleviate this problem.

In the course of developing our software, we had to implement a connection to the laboratory hardware we use. The interfaces provided by the manufacturers for this purpose are not very powerful and rather user-unfriendly. More powerful, well-documented and standardized interfaces for controlling laboratory hardware would be highly desirable in the future.

Using our system requires basic programming skills. The internals of the framework are encapsulated in classes that do not have to be edited or understood by the average user, but for defining new experiment types or parameterizing existing ones, small R-scripts have to be written or edited. We found that users without prior programming experience are able to use and modify a “construction kit” of existing scripts after basic training, but debugging and dealing with unexpected situations needs a lab-member with some computer-science skills. As a solution, specific applications that are needed for routine-purposes could be “hidden” under a graphical user interface that allows only editing of the necessary parameters. Another, more flexible, but less simple approach would be an easy formal language for describing experimental protocols that can be translated into hardware instructions automatically by specialized compilers.

Despite these limitations, we are convinced that computer controlled experimenting like that provided by our framework provides a powerful, convenient and flexible way of using lab automation equipment for innovative applications in method development and systems biology.

## Materials and Methods

### System and Software

Our hardware setup consists of a Tecan Genesis RSP 159 liquid handling robot and a Tecan Ultra II plate reader. The robot is equipped with eight 1000µl Teflon-coated nondisposable steel pipette tips and a gripper that can be used for transporting standard microtiter-plates between the robot deck and the reader located by its side. Both devices are controlled by R scripts, which use a set of in-house R packages developed for this purpose. Pipetting commands are passed to the robot by executing program-generated worklists using the “named-pipe” interface of the Tecan Gemini robot-control software. The plate reader is controlled using an in-house modified version of the XFluor Excel macros supplied by the manufacturer for manual measurements. The modifications carried out by us allow configuring, executing and exporting measurements without user-interactions.

Our R packages provide an abstraction layer for general fluid mixture based experiments that allows carrying out variants of an experiment by providing a table with concentrations of each mixture component. Component volumes are calculated from the concentrations and all necessary pipetting and measurement steps are generated and executed automatically.

### Interpolation and smoothing based on Gaussian Random Process Regression

In order to visualize the multidimensional datasets that result from assays with different mixture compositions, we needed a method that provides two-dimensional interpolated slices through the multidimensional parameter space.

The framework of Gaussian Random Process Regression provides a powerful method for interpolation and smoothing of noisy datasets [12]. It has been studied intensively for applications in geostatistics, where it is often called *Kriging* [13]. Gaussian Random Process Regression is based on the general assumption that measurements at points that are close to each other in the parameter-space co-vary in a way that can be described by some covariance function. The form of the covariance function and its parameters such as the maximum covariance, its characteristic length-scale and the intrinsic noise of the measurements can be estimated by maximum-likelihood or cross-validation, making it a very flexible technique for regression without strong a-priori-assumptions.

We developed an extension of the Gaussian Random Process Regression implementation in the *fields* R package for visualizing slices of multidimensional datasets and obtaining nonparametric surrogate models of experimental systems that can be used for optimization [14], [15]. The *fields* implementation uses generalized cross validation to obtain an estimate of the noise-level in the data and find an optimal smoothing parameter. In addition, our implementation performs an optimization of the length-scale parameter of the covariance function in all dimensions of the model using the Nelder-Mead method as implemented in the R function *optim* with cross-validation results of *fields* as the objective function. Our implementation allows scaling of the axes with arbitrary functions that reflect a-priori assumptions about the sensitivity of the system to parameter changes in different regions of the parameter space. This allows switching to a logarithmic scaling of certain axes, which is useful for many biochemical systems that show a large variability at low concentrations.

### Optimization of Experimental Parameters

We implemented a response-surface-model based algorithm for optimizing parameters of experimental systems that uses smooth surrogate models of the system behaviour obtained by Gaussian Random Process Regression [14], [15]. While optimization based on noisy measurements is hard, the smooth surrogate models can be optimized using conventional quasi-Newton methods such as those provided in R. The algorithm is formulated as a minimization of an objective function defined by the user. When maximization is desired (i.e. of enzymatic activity in a buffer system), the sign of the objective function can simply be reversed.

In a first round of experiments, our algorithm constructs a coarse initial model of the system behaviour with a relatively low number of measurements distributed evenly in the feasible region of the parameter space. A suitable space-filling experimental design is generated by the algorithm. After fitting the initial model, it can be refined with additional rounds of measurements with parameters chosen by the algorithm. The new measurement points are chosen to lie in those regions where the lower bound of the model estimate is lowest, while keeping a user-specified minimum distance to other measurements (similar to method 3 in [14]). Specifically, parameters for a new round of experiments are obtained by sampling from a large number of randomly generated candidate points. The probability of choosing a point follows Boltzmann-distribution with the lower bound of the model-estimate as “Energy”.

If a point violates the minimum distance requirement, a large number of alternative points is generated from a multinomial normal distribution centred at the candidate point. From these points, the one that is closest to the original point while maintaining the distance requirement is selected. If none of the new points satisfies the distance requirement, the one that is farthest away from other points is selected.

If the reagents are stable enough to allow multiple experiments to be carried out in a row using the same stocks, the complete optimization process including multiple rounds of model-refinement can be performed automatically and without requiring user-interaction.

### Enzymatic Assays

Crude extracts were prepared from 20 OD_600_ units of exponentially growing *Saccharomyces cerevisiae* wild-type cells (FF18984 strain: *MATa leu2-3,112 ura3-52, lys2-1, his7-1*) with 1 g acid-washed glass beads (0.4–0.5 mm diameter) in 0.5 ml 20 mM Hepes, pH 7.1, 100 mM KCl, 5 mM MgCl2, 1 mM EDTA and 1 mM DTT. All procedures were carried out at 0–4°C. Samples were vortexed (3×5 min with cooling on ice in between) in Mixer Mill MM 300 (Retsch). After centrifugation at 16000 g for 15 min at 4°C, the supernatant was immediately used for enzymatic assays. Protein content was determined by the method of Bradford [16]. All chemicals and enzymes for enzymatic assays were purchased from Sigma.

Activity of pyruvate kinase (PYK, EC 2.7.1.40) in yeast lysate was measured by coupling production of pyruvate from phospho(enol)pyruvate and ADP to the consumption of NADH [17]. NADH consumption was monitored by kinetic absorption measurements at 340 nm in the thermostatted reader at 30°C. Lactic dehydrogenase (LDH, EC 1.1.1.27) acted as the coupling enzyme and was added in extreme excess in order to minimize its influence on the measured activities. The pH of the reaction mixture was adjusted by adding different fractions of two TRIS/MES pH-buffers adjusted to different pH-values. The necessary volumes were calculated from a spline-fit of pH measurements of 16 different buffer mixtures. The reaction mixture consisted of 62.5µl TRIS/MES pH-buffer mixture (50mM, pH 4.75–9.2), 0–30µl KCl (0–266.6 mM), 0–40µl Fructose 1,6-bisphosphate (0–10mM), 10µl NADH (0.625mM), 0–45µl PEP (0–8mM), 10µl ADP+MgCl_2_ (4.6875 mM+5mM), 10µl Enzyme/lysate mixture (0.2 µg total protein+0.65 units of lactic dehydrogenase (EC 1.1.1.27) per reaction) and H_2_O to bring the mixture to 225µl end volume.

All reaction components except reaction starter (ADP) were pipetted into the wells of a 96 well plate (black, flat UV-transparent bottom, Greiner) individually using single-dispense-pipetting for maximum pipetting precision. The most stable components (pH buffers, salt solutions and water) of the mixture were added first, in order to avoid transient exposure of the less stable components (cofactors, substrates, enzymes) to extreme conditions.

After pipetting of these components was finished, the plate was transferred to the plate-reader using the robotic gripper and incubated there for 10 minutes in order to enable temperature equilibration to 37°C at which the assay was performed and to expose the enzymes to the final conditions for a defined minimum amount of time.

After incubation, the reaction was started by adding ADP. Multiple dispense pipetting was used to reduce the time spent with pipetting while the reaction in the first wells is already running. After transferring the plate into the reader, an absorption-kinetics measurement with 30 measurement cycles was started by our program. The kinetic cycle interval was set to minimum (approx. 60s for 96 wells) and only the wells that were actually used in the experiment were measured for maximum time resolution. The final measurement time was between 15 min and 30 min with kinetic intervals between 30s and 60s. The slope of the kinetic curves was determined by linear regression in a region selected by a combination of minimum and maximum OD values and measurement times.

The initial reaction speed and thus the enzymatic activity in each well (*act_total_*) was calculated by dividing the slope of the kinetic curve (*A_340_/dt*) by the plate-specific molar-absorption coefficient of NADH (*ε_NADH_*).
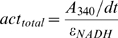
The constant *ε_NADH_* was determined from a standard curve of the *A_340_* values of 150 µl of 1 mM, 0.5 mM, 0.25 mM and 0.125 mM NADH in the wells of a 96 well plate of the same type used for our enzymatic assays. Samples containing all reaction components, excluding the coupling enzyme LDH, at various pH conditions were used as controls for passive oxidation rate of NADH. The changes in background signal are much smaller than changes observed in the coupled reaction, showing a small change at pH<5.5 in the NADH oxidation rate that does not significantly influence the coupled reaction (Figure S1).

The activity per mg protein content (act_permgprotein_) was obtained by dividing *act_total_* by the amount of protein in the well as determined from the extinction in a Bradford test (*A_BF_*) and the lysate volume (*V_lysate_*).
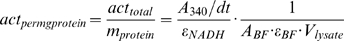
(1)The Bradford-test proportionality constant *A_BF_* was determined from a standard curve.

### pH buffer system

**Figure 9:**
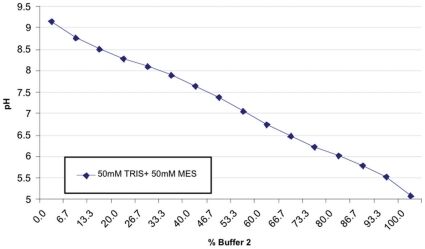
The calibration curve used for converting between buffer-fractions and pH values. The measured pH values of 16 different mixtures are shown as dots. Notice the nearly linear dependence between buffer-composition and pH in the range between pH 8.5 and pH 5.5.

The pH in the reaction mixtures was controlled by adding mixtures of two buffers adjusted to different pH values. The same buffer system was used for both buffers and the total buffer volume was kept constant. In order to be able to vary the pH in the reaction reproducibly over the desired range, we used a two component system of 2-(N-morpholino)ethanesulfonic acid (MES, pKa = 6.15) and tris(hydroxymethyl)aminomethane (TRIS, pKa = 8.06) in equal concentrations of 50mM. The pH of stock mixtures was adjusted by addition of NaOH or HCl to pH 9.2 and pH 4.75, respectively. The two buffer stocks were mixed in different ratio providing nearly linear dependence of the pH on the mixture composition (see Fig. 9). We used a spline fit of the data shown in the figure for converting between buffer fractions and pH values.

### A diprotic model of pH dependence of PYK activity

**Figure 10:**
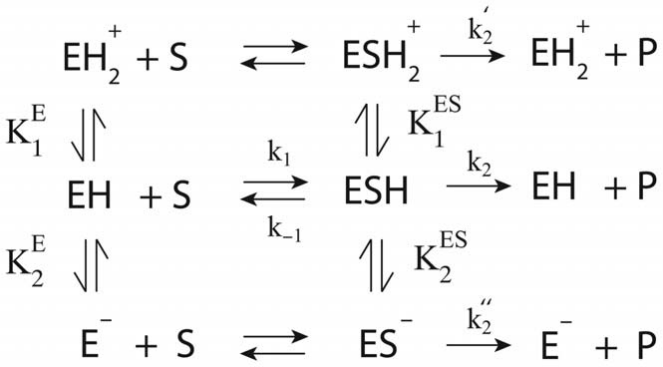
Reaction scheme of diprotic model. E represents the enzyme as a weak diprotic acid with its three protonation states EH_2_^+^, EH and E^−^. The dissociation constants K_1_^E^, K_2_^E^, K_1_^ES^ and K_2_^ES^ describe rapid protonization equilibria of the free enzyme and the enzyme with bound substrate, i.e., K_1_^E^ = [H^+^][EH]/[EH_2_^+^], K_2_^E^ = [H^+^][E^−^]/[EH], K_1_^ES^ = [H^+^][ESH]/[ESH_2_^+^], K_2_^ES^ = [H^+^][ES^−^]/[ESH]. For the sake of simplicity, free protons are not shown in the scheme. The substrate affinity of the neutral enzyme EH is given by K_M_ = [EH][S]/|ESH]. In case of steady-state kinetics, K_M_ = (k_−1_+k_2_)/k_1_. The reaction rate of the system is given as d[P]/dt = k_2_[ESH]+k_2_′[ESH_2_^+^]+k_2_″[ES^−^] leading to the final expression described by Eq. 2.

For modeling the pH dependence of PYK activity, we used a classical diprotic model of enzymatic activity [18], [19], [20], [21]. A sketch of the reaction network and the model parameters is shown in Fig. 10 and Table 2. The model treats the enzyme like a weak diprotic acid with two protonation-sites. Depending on its protonation state, the enzyme has different substrate affinities and speeds of the rate limiting substrate conversion step, resulting in a pH dependent transition between different Michelis-Menten like substrate saturation behaviours. Note that in this model the proteolytic properties of the substrate (PEP) are not considered.

**Table 2:** Parameters of the diprotic model of enzymatic activity as determined by nonlinear least squares regression.

Parameter name	Value from nonlinear least squares
V_max_	1.1•10^4^ mU
K_M_	0.53 mM
K_1_^E^	1.9 •10^−6^ M
K_2_^E^	4.4 •10^−9^ M
K_1_^ES^	2.2 •10^−6^ M
K_2_^ES^	9.6 •10^−9^ M
α	0.16
β	0

For the meaning of the parameters, see Fig. 10.

A detailed derivation of the formula used for calculating enzymatic activities from substrate concentrations and pH values can be found in the literature [18], so we give the final result only. The enzymatic activity/rate is calculated as follows:
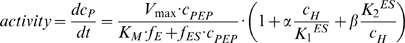
(2)where *c_H_* and *c_P_* are proton and product P concentrations, respectively. The proton concentration *c_H_* is related to the pH as follows:
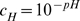
(3)The two terms *f_E_* and *f_ES_* denote the (Michaelis) acidity functions:
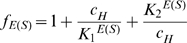
(4)α and β are ratios between turnover-numbers 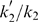 and 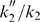, respectively (Fig. 10, Table 2). The presence of α values indicate that the acidic form of the enzyme (EH_2_^+^) is catalytically active, while the presence of β values indicate that the basic form of the enzyme (E^−^) is catalytically active.

The parameters of the model were determined by minimizing the sum of squared differences between model predictions and measured data using the R function *nls*. A manual guess of the parameters was used as a starting point. To ensure that a global optimum was found, the fitting procedure was initialized with 5000 different parameter combinations derived from the manual estimate by multiplying each parameter with a random number between 10^−3^ and 10^3^. Among those fits that converged, we selected the one with the lowest sum of squared residuals.

The *optimum pH* refers to the pH values, which at constant substrate concentrations leads to the largest rate/activity in the enzymes and refers to the maximum of the bell-shaped activity-pH plot. It should not be confused with the V_max_ of the enzyme (Eq. 2). Using the diprotic model with α = β = 0, the optimum proton concentration 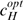 can be estimated from Eq. 2 by finding the maximum activity, i.e., calculating the derivative of the activity with respect to *c_H_*, setting it to zero and solving for 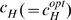:
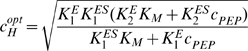
(5)


## Supporting Information

Figure S1 Influence of pH and KCl concentration in reaction buffer on spontaneous NADH oxidation. Each dot corresponds to a measurement conducted with one of 48 conditions from the space filling design of the PYK-LDH coupled assay. In all plots, the measured data and the Kriging model fitted surface is shown. a) Blue response surface represents samples without the coupling enzyme LDH; red surface represents samples whith all reaction components present (PEP = 1 mM, see Material and Methods). b) Extended view of the response surface model of spontaneous NADH oxidation in samples where LDH is excluded from the reaction (blue response surface in a); but extended scale of z-axes to visualize changes in spontaneous NADH oxidation). Results show a low spontaneous oxidation of NADH at pH<5.5, which is significantly lower than the changes resulting from enzymatic reactions.
(1.95 MB TIF)
