# Supplementary figures and images for: Computer Controlled Automated Assay for Comprehensive Studies of Enzyme Kinetic Parameters
Source: PLoS One. 2010 May 19;5(5):e10727. doi: 10.1371/journal.pone.0010727 (PMC2873295; doi:10.1371/journal.pone.0010727)

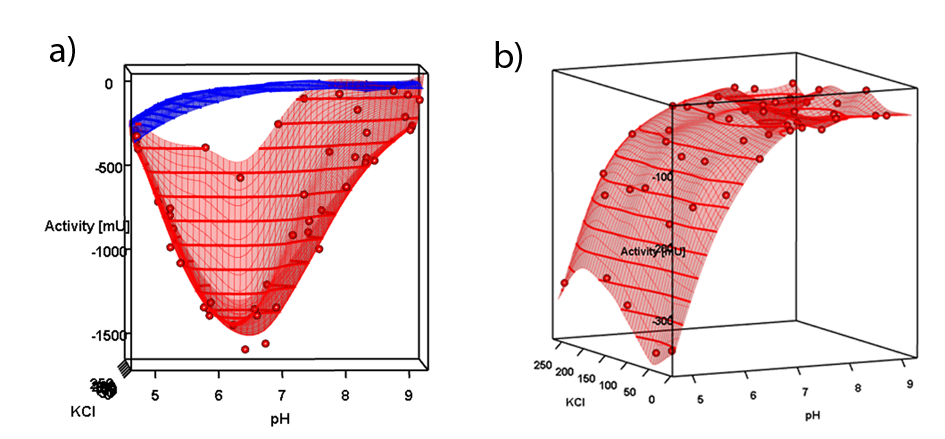

Supplement: Figure S1 — Influence of pH and KCl concentration in reaction buffer on spontaneous NADH oxidation. Each dot corresponds to a measurement conducted with one of 48 conditions from the space filling design of the PYK-LDH coupled assay. In all plots, the measured data and the Kriging model fitted surface is shown. a) Blue response surface represents samples without the coupling enzyme LDH; red surface represents samples whith all reaction components present (PEP = 1 mM, see Material and Methods). b) Extended view of the response surface model of spontaneous NADH oxidation in samples where LDH is excluded from the reaction (blue response surface in a); but extended scale of z-axes to visualize changes in spontaneous NADH oxidation). Results show a low spontaneous oxidation of NADH at pH<5.5, which is significantly lower than the changes resulting from enzymatic reactions. (1.95 MB TIF) [file pone.0010727.s001.tif]
